# Circ_0008039 supports breast cancer cell proliferation, migration, invasion, and glycolysis by regulating the miR‐140‐3p/SKA2 axis

**DOI:** 10.1002/1878-0261.12862

**Published:** 2020-12-07

**Authors:** Dongwei Dou, Xiaoyang Ren, Mingli Han, Xiaodong Xu, Xin Ge, Yuanting Gu, Xinxing Wang, Song Zhao

**Affiliations:** ^1^ Department of Breast Surgery The First Affiliated Hospital of Zhengzhou University China; ^2^ Department of Information The First Affiliated Hospital of Zhengzhou University China; ^3^ Department of Thoracic surgery The First Affiliated Hospital of Zhengzhou University China

**Keywords:** breast cancer, circ_0008039, miR‐140‐3p, SKA2

## Abstract

Circular RNAs (circRNAs) have been shown to modulate gene expression and participate in the development of multiple malignancies. The purpose of this study was to investigate the role of circ_0008039 in breast cancer (BC). The expression of circ_0008039, miR‐140‐3p, and spindle and kinetochore‐associated protein 2 (SKA2) was detected by qRT‐PCR. Cell viability, colony formation, migration, and invasion were evaluated using methylthiazolyldiphenyl‐tetrazolium bromide (MTT) assay, colony formation assay, and transwell assay, respectively. Glucose consumption and lactate production were measured using commercial kits. Protein levels of hexokinase II (HK2) and SKA2 were determined by western blot. The interaction between miR‐140‐3p and circ_0008039 or SKA2 was verified by dual‐luciferase reporter assay. Finally, a mouse xenograft model was established to investigate the roles of circ_0008039 in BC *in vivo*. We found that circ_0008039 and SKA2 were upregulated in BC tissues and cells, while miR‐140‐3p was downregulated. Knockdown of circ_0008039 suppressed BC cell proliferation, migration, invasion, and glycolysis. Moreover, miR‐140‐3p could bind to circ_0008039 and its inhibition reversed the inhibitory effect of circ_0008039 interference on proliferation, migration, invasion, and glycolysis in BC cells. SKA2 was verified as a direct target of miR‐140‐3p and its overexpression partially inhibited the suppressive effect of miR‐140‐3p restoration in BC cells. Additionally, circ_0008039 positively regulated SKA2 expression by sponging miR‐140‐3p. Consistently, silencing circ_0008039 restrained tumor growth via increasing miR‐140‐3p and decreasing SKA2. In conclusion, circ_0008039 downregulation suppressed BC cell proliferation, migration, invasion, and glycolysis partially through regulating the miR‐140‐3p/SKA2 axis, providing an important theoretical basis for treatment of BC.

## Graphical abstract

This study describes the role of circ_0008039 in breast cancer. We found that high expression of circ_0008039 was significantly associated with lymph node metastasis and tumor stage of breast cancer patients, and circ_0008039 knockdown could inhibit breast cancer cell proliferation, invasion, and glucose metabolism by upregulating miR‐140‐3p and downregulating SKA2.

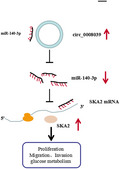

**Abbreviations**
ANOVA: analysis of variance
BC: breast cancer
circRNAs: circular RNAs
DMSO: dimethyl sulfoxide
ECAR: extracellular acidification rate
ECL: enhanced chemiluminescence
FBS: fetal bovine serum
HK2: hexokinase II
MEGM: mammary epithelial growth medium
miR‐140‐3p: microRNA‐140‐3p
MTT: methylthiazolyldiphenyl‐tetrazolium bromide
PBS: phosphate‐buffered saline
PRKAR1B: protein kinase A regulatory subunit R1‐beta
SD: standard ± deviation
SKA2: spindle and kinetochore‐associated protein 2

## 1 Introduction

Breast cancer (BC) is the most commonly diagnosed cancer among women and the leading cause of cancer death, with about 2.1 million new cases in 2018 [1]. Although a great number of target treatments have been developed in the past few decades, the prognosis of BC patients with distant metastatic cancer is still unsatisfactory [2]. Hence, it is imperative to explore the pathogenesis of BC and thus developing more effective therapeutic targets for BC treatment.

Circular RNAs (circRNAs) are a special type of noncoding RNAs (ncRNAs) without 5’‐end cup and 3’‐end ploy A tail and play key roles in regulating the occurrence and development of diverse diseases [3, 4]. Compared to linear RNAs, circRNAs have higher tolerance to RNaseR exonuclease and are more stable and difficult to degrade [5]. Recently, the development of sequencing technology has led to the identification of multiple circRNAs in diverse cell types [6]. Accumulating evidence has demonstrated that BC progression can be regulated by several circRNAs, such as circ‐ABCB10 [7], circ_001982 [8], and circ_0011964 [9]. Hsa_circ_0008039 is derived from back‐splicing of protein kinase A regulatory subunit R1‐beta (PRKAR1B) transcript and located at chr7:716865‐751164, and circ_0008039 has been suggested to act as a tumor facilitator in BC [10]. However, the biological functions and regulatory mechanism of circ_0008039 are still largely unknown in BC.

CircRNAs commonly exert their functional roles via serving as microRNA (miRNA) inhibitors or sponges in different cancers [11]. MiRNAs are a class of ncRNAs (~ 22 nucleotides) that negatively modulate the expression of target genes via directly binding to the 3’UTR of target mRNAs, and miRNAs were reported as key regulators in multiple cancers [12, 13]. Previous reports have shown that miR‐140‐3p usually serves as a tumor‐suppressive miRNA in some cancers, including BC [14, 15]. Nevertheless, the association between circ_0008039 and miR‐140‐3p in BC remains unclear.

Spindle and kinetochore‐associated protein 2 (SKA2) was identified as a tumor promoter and participated in various critical cellular mechanisms, including cell growth, metastasis, cell cycle, and so on [16, 17]. Moreover, previous research proved that SKA2 was highly expressed in BC tissues, and its downregulation repressed BC cell proliferation, migration, and invasion [18]. However, the circ_0008039/miR‐140‐3p/SKA2 regulatory network in BC has not been reported.

Herein, the expression levels of circ_0008039, miR‐140‐3p, and SKA2 were examined in BC tissue samples and cell lines. Besides, we studied the regulatory network of circ_0008039/miR‐140‐3p/SKA2 in BC and also assessed their effects and underlying mechanisms in BC, which might offer a new mechanism and therapeutic strategy for BC.

## 2 Materials and methods

### 2.1 Clinical samples

BC tissues (*n* = 51, 24 primary tumor specimens (stages I/II) and 27 advanced tumor specimens (stages III/IV)) and adjacent normal tissues (*n* = 51) were acquired from patients who underwent surgery at the First Affiliated Hospital of Zhengzhou University. The subtype of breast cancer we studied is triple negative breast cancer (TNBC). Staging was assessed in line with the International Union Against Cancer’s (UIAC) tumor‐node metastasis (TNM) system. All patients did not undergo any therapy before the operation and signed informed consents prior to surgery. These tissue specimens were collected and then timely frozen in liquid nitrogen until the experiments were performed. Tissue collection and experiments were performed in compliance with the Helsinki Declaration and were approved by Research Ethics Committee of the First Affiliated Hospital of Zhengzhou University.

### 2.2 Cell culture and transfection

BC cell lines BT20, BT549, MDA‐MB‐231 (MB‐231), and MDA‐MB‐468 (MB‐468) and normal human breast epithelial cells (MCF10A) were bought from BeNa Culture Collection (Beijing, China). MCF‐10A cells were allowed to grow in mammary epithelial growth medium (MEGM; Lonza Clonetics, Walkersville, MD, USA) supplemented with 100 ng·mL^−1^ cholera toxin (Sigma‐Aldrich, St. Louis, MO, USA). The BC cell culture medium was the RPMI‐1640 medium (Invitrogen, Carlsbad, CA, USA) with 10% fetal bovine serum (FBS; Invitrogen). All cells were maintained at constant temperature incubator with 5% CO_2_ at 37 °C.

The small interfering RNA (siRNA) against circ_0008039 or SKA2 (si‐circ_0008039 or si‐SKA2) and matched control (si‐NC), miR‐140‐3p mimic or inhibitor (miR‐140‐3p or anti‐miR‐140‐3p) and matched control (miR‐NC or anti‐miR‐NC), SKA2 or circ_0008039 overexpression plasmid (SKA2, circ_0008039), and matched control (vector) were obtained from RiboBio (Guangzhou, China). Lentivirus‐mediated shRNA interference targeting (sh‐circ_0008039) and its negative control (sh‐NC) constructed by GeneCopoeia (Rockville, MD, USA). For cell transfection, MB‐231 and MB‐468 cells with 60–70 confluences were transfected with oligonucleotide or vector using Lipofectamine 3000 (Invitrogen).

### 2.3 Subcellular fractionation location

Referring to manufacturer’s instructions, PARIS Kit (Life Technologies Corp., Grand Island, NY, USA) was used for separating cytoplasmic and nuclear RNAs. Next, the expression of circ_0008039, U6, and GAPDH was gauged via qRT‐PCR analysis. GAPDH or U6 was served as a control for the cytoplasm or nucleus, respectively.

### 2.4 Characterization of circ_0008039 in BC cells

To verify the circular character of circ_0008039, oligo (dT) and random primers were used in the reverse transcription experiments. To examine the stability of circ_0008039 and corresponding linear mRNA, RNA sample (5 μg) was incubated without RNase R or with 3 U·μg^−1^ RNase R (Epicentre Technologies, Madison, WI, USA) at 37 °C for 0.5 h. Actinomycin D (2 mg·mL^−1^) or dimethyl sulfoxide solution (DMSO; Sigma‐Aldrich) was added to cultured medium, followed by RNA extraction. At last, the abundance of circ_0008039 and PRKAR1B was tested using qRT‐PCR analysis.

### 2.5 RNA extraction and qRT‐PCR

Referring to instruction of manufacturers, TRIzol reagent (Invitrogen) was applied for isolating total RNA from tissues or cell lines. For detection of gene expression levels, cDNA was synthesized using a Prime Script RT reagent Kit (Takara, Dalian, China). Then, the diluted cDNA was subjected to qRT‐PCR using the SYBR Green Master Mix (Applied Biosystems, Foster, CA, USA) on 7500 Real‐time PCR System (Applied Biosystems). GAPDH (for circ_0008039, linear PRKAR1B and SKA2) and U6 (for miR‐140‐3p) were used as references. RNA relative level was evaluated by 2^−ΔΔCt^ method. Primer sequences: circ_0008039 (F, 5’‐AACGTGCTCTTCGCTCACCT‐3’; R , 5’‐CGTACAGCTCACAGCCCTTCA‐3’), linear PRKAR1B (F, 5’‐GTGAGTGCCGAGGTGTAC‐3’; R 5’‐CATCCAGGTGAGCGAAGAG‐3’), miR‐140‐3p (F, 5’‐TCGGCAGGTAACACTGTCTGGT‐3’; R , 5’‐CTCAACTGGTGTCGTGGA‐3’), pri‐miR‐140‐3p (F, 5’‐TGGTGTGTGGTTCTATGCCAGC‐3’; R, 5’‐CTCAAGCCAGAATTCAGG‐3’), pre‐miR‐140‐3p (F, 5’‐CCTGCCGTGGTTTTACCCT‐3’; R, 5’‐AGGGTAGAACCACGGCAGG); SKA2 (F, 5’‐CTGAAACTATGCTAAGTGGGGGAG‐3’; R 5’‐TTCCAAACATCCTGACACTCAAAAG‐3’) GAPDH (F, 5’‐CGCTCTCTGCTCCTCCTGTTC‐3’; R, 5’‐ ATCCGTTGACTCCGACCTTCAC‐3’), U6 (F, 5’‐CTCGCTTCGGCAGCACATATACT‐3’; R, 5’‐ACGCTTCACGAATTTGCGTGTC‐3’).

### 2.6 Methylthiazolyldiphenyl‐tetrazolium bromide (MTT) assay

MTT assay was used for measuring MB231 and MB468 cell viability. In short, MB231 and MB468 cells (5 × 10^3^ cells each well) were inoculated into 96‐well plates. After transfection, MTT reagent (5 mg·mL^−1^, 20 μL, Beyotime, Shanghai, China) was added to per well using a pasteur pipette (20 μL), followed by incubation for 3–4 h at 37 °C. Next, the cultured medium was carefully removed using a pasteur pipette (200 μL), followed by addition of DMSO (150 μL, Sigma‐Aldrich) to dissolve formazan. Finally, cell viability was determined via detecting the absorbance using a microplate reader (Bio‐Teck, Winooski, VT, USA) at 490 nm.

### 2.7 Colony formation assay

In brief, MB231 and MB468 cells were inoculated into six‐well plates. After 48 h of transfection, MB231 and MB468 cells were cultured in fresh medium and the medium should be updated every 3 days during culture. After 14 days, MB231 and MB468 cells were gently washed with PBS, followed by fixing with paraformaldehyde (4%, Beyotime) for 0.5 h at 4 °C. Then, these cells were again washed by PBS, followed by staining with crystal violet (0.1%, Beyotime) for 2 h. A microscope (Olympus, Tokyo, Japan) was employed for counting the colonies (> 50 cells per colony).

### 2.8 Transwell assay

Migration and invasion of MB231 and MB468 cells were measured with transwell chamber (8 μm pore, Costar, Corning, NY, USA). For invasion assay, the transwell chambers were precoated with Matrigel (BD Biosciences, San Jose, CA, USA). The top chambers were added with cell suspension in serum‐free medium (0.2 mL). Meanwhile, the bottom chambers were filled with complete medium (0.6 mL). At 24 h postincubation, the lower chambers with filtered cells were immobilized with paraformaldehyde (4%, Beyotime) and stained with crystal violet (0.1%, 1 h), and then imaged with a microscope. For migration assay, MB231 and MB468 cells were inoculated into the top chambers without a Matrigel coating, and the other procedures were same to the invasion assay.

### 2.9 Measurement of glucose consumption, lactate production, and extracellular acidification rate (ECAR)

Referring to instruction of manufacturers, a glucose assay kit (Sigma‐Aldrich) and a lactate assay kit (BioVision, Mountain View, CA, USA) were applied for measuring the glucose consumption and lactate production, respectively. The data were determined with a standard calibration curve and normalized to the amount of total protein. In addition, ECAR was measured by using Seahorse XF Glycolysis Stress Test Kit (Agilent, Santa Clara, CA, USA) following the XF glycolysis stress test protocol.

### 2.10 Western blot assay

RIPA lysis buffer (Thermo Fisher, Wilmington, DE, USA) was utilized to extract the total protein. Then, the protein was added to 2 × loading buffer (Beyotime) and boiled for degeneration, followed by quantification with BCA protein assay kit (Tanon, Shanghai, China). Next, 10–12% SDS/PAGE was applied to separate the protein, and the protein was transferred onto PVDF membranes (Beyotime). Then, the membranes would be blocked in nonfat milk (5%, Sangon Biotech, Shanghai, China) for 1.5–2 h, followed by incubation with primary antibody against Hexokinase II (HK2) (ab227198, 1:5000, Abcam, Cambridge, UK), SKA2 (ab735345, 1:1000, Abcam) or GAPDH (ab37168, 1:2000, Abcam) for 12–16 h at 4 °C. After that, the corresponding secondary antibody (D110058, 1:4000, Sangon Biotech) was utilized for the combination with the primary antibody, and the combined signals were examined using the enhanced chemiluminescence (ECL) reagent (Tanon). Protein expression levels (HK2 and SKA2) were evaluated using ImageJ software, and GAPDH was employed as a reference.

### 2.11 Dual‐luciferase reporter assay

The binding sequences of miR‐140‐3p and circ_0008039 or SKA2 were provided by Circular RNA Interactome or starBase v2.0. The wild‐type (WT) sequence of circ_0008039 or SKA2 3’UTR with binding sites for miR‐140‐3p was individually inserted into pmirGlO luciferase reporter vector (Promega, Madison, WI, USA) to create WT plasmids (WT‐circ_0008039, WT‐SKA2). Meanwhile, their mutant (MUT) plasmids (MUT‐circ_0008039, MUT‐SKA2) without binding sequence for miR‐140‐3p were generated in the same way. MB231 and MB468 cells were cotransfected with above reporter plasmid and miR‐140‐3p or its control (miR‐NC). Following 48 h of cotransfection, dual‐luciferase reporter assay system (Promega) was employed for measuring the luciferase activities.

### 2.12 Tumor xenograft model

MB468 cells (1 × 10^6^) transiently transfected with sh‐NC (as control) or sh‐circ_0008039 were subcutaneously injected into female BALB/c nude mice (weighing 18–20 g, 5 weeks old, *n* = 5/group, Shanghai Experimental Animal Center, Shanghai, China). The xenograft mice experiments obtained the approval from the Committee of Animal Research of the First Affiliated Hospital of Zhengzhou University. Tumor size was examined every week using slide calipers and calculated based on the formula: volume = length × width^2^ × 0.5. 4 weeks later; all mice would be sacrificed, and then, tumor samples were used for weight assessment and further analysis.

### 2.13 Statistical analysis

All data were presented as standard ± deviation (SD) from at least three independent experiments. Student’s *t*‐test was used to compare the differences between two groups. Multiple (> 2) group comparison was analyzed with a one‐way analysis of variance (ANOVA). Spearman’s correlation tests were applied to analyze the association between miR‐140‐3p and circ_0008039 or SKA2. GraphPad Prism software ver. 5.0 was used for data analysis. Differences were considered significant when the *P* < 0.05.

## 3 Results

### 3.1 Identification and characteristics of circ_0008039 in BC cells

**Fig. 1:**
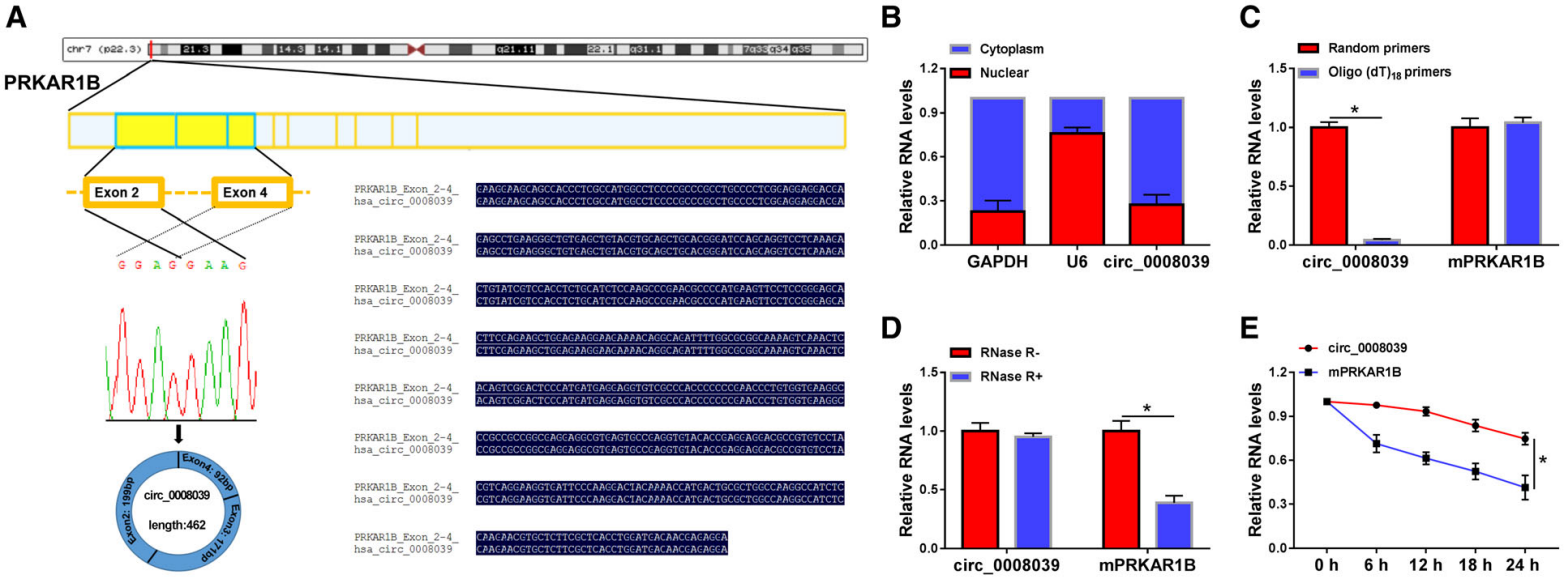
Validation and characteristics of circ_0008039 in BC cells. (A) The diagram shows that circ_0008039 is generated from exons 2–4 of the PRKAR1B gene. (B) The qRT‐PCR assay was used to determine the subcellular location of circ_0008039 in MB231 cells. (C) The levels of circ_0008039 and PRKAR1B mRNA were detected after reverse transcription with random or oligo (dT)_18_ primers via qRT‐PCR. (D and E) After treatment with actinomycin D (D) and RNase R (E), circ_0008039 and PRKAR1B mRNA expression levels were examined using qRT‐PCR in MB231 cells. The data are shown as the means ± SD of 3 independent experiments. Statistical analysis was conducted using Student’s *t*‐test. *P < 0.05.

In accordance with Circular RNA Interactome, the circ_0008039 is generated from exons 2–4 of PRKAR1B gene and is located on chromosome 7 (Fig. 1A). Cytoplasmic and nuclear RNA analysis demonstrated that circ_0008039 was primarily located in the cytoplasm (Fig. 1B). Moreover, qRT‐PCR analysis proved that circ_0008039 level was obviously lower when using oligo (dT)_18_ primers than when using random primers (Fig. 1C), suggesting that absence of a poly (A) tail for circ_0008039. We then assessed the stability of circ_0008039. Compared with its linear mRNA (PRKAR1B), circ_0008039 was resistant to RNase R (Fig. 1D), suggesting the cyclic structure of circ_0008039. At the same time, the results of actinomycin D assay indicated that the half‐life of circ_0008039 transcript could exceed 24 h, while that of linear PRKAR1B exhibited only about 18 h, suggesting that circ_0008039 transcript was more stable compared to linear PRKAR1B mRNA transcript (Fig. 1E). Overall, our data suggested that the circ_0008039 was stable and had a closed‐loop structure.

### 3.2 Circ_0008039 was overexpressed in BC tissues and cells

**Fig. 2:**
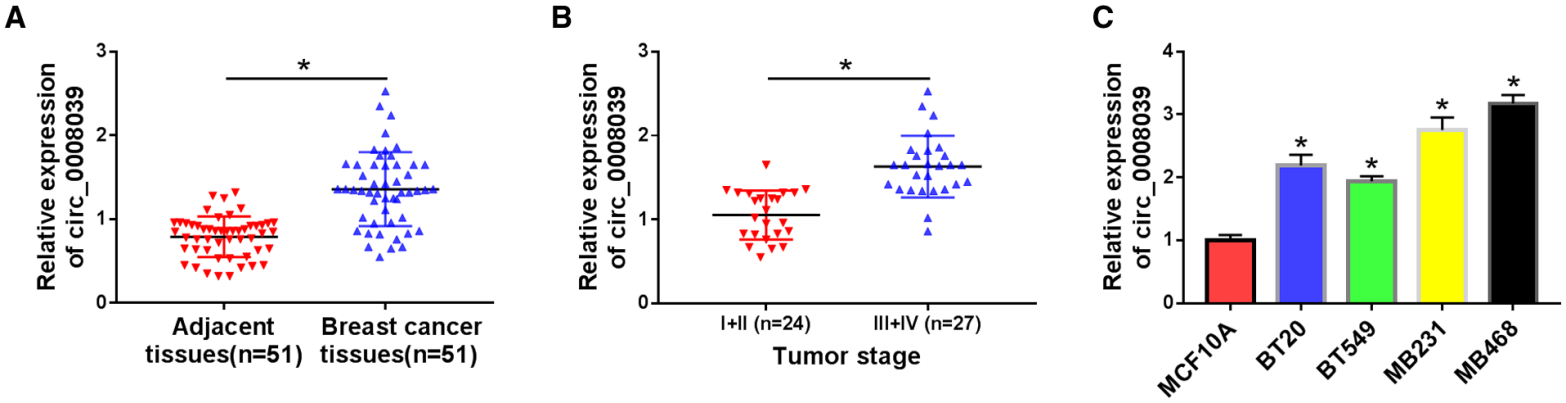
The expression of circ_0008039 was enhanced in BC tissues and cells. (A) The expression of circ_0008039 was detected in 51 pairs of BC tissues and adjacent normal tissues. (B) Circ_0008039 level was measured in patients with pathological stages I + II (*n* = 24) and pathological stage III + IV (*n* = 27). (C) Circ_0008039 level was detected in BC cells (BT20, BT549, MB231, and MB468) and MCF10A cells. The data are shown as the means ± SD of 3 independent experiments. Statistical analysis was conducted using Student’s *t*‐test or ANOVA. *P < 0.05.

To explore circ_0008039 expression in BC, BC tissues (*n* = 51) and corresponding normal tissues (*n* = 51) were collected and qRT‐PCR was carried out. As displayed in Fig. 2A, circ_0008039 was expressed at a high level in BC tissues in contrast to adjacent normal tissues. Furthermore, we uncovered that circ_0008039 level was markedly higher in patients with pathological stages III/IV (*n* = 27) than those with pathological stages I/II (*n* = 24) (Fig. 2B). Moreover, the relationship between circ_0008039 expression and clinical features in BC patients was assessed. As exhibited in Table S1, high level of circ_0008039 was positively correlated with tumor stage and lymph node metastasis, but its expression was not correlated with age, tumor size, and menopause. Increased expression of circ_0008039 was also observed in BC cells (BT20, BT549, MB231, and MB468) compared to MCF10A, especially in MB231 and MB468 cells (Fig. 2C). Therefore, we chose MB231 and MB468 cells for the following experiments. Our data revealed that circ_0008039 might play a carcinogenic role in BC.

### 3.3 Knockdown of circ_0008039 repressed the proliferation and malignant features of BC cells

**Fig. 3:**
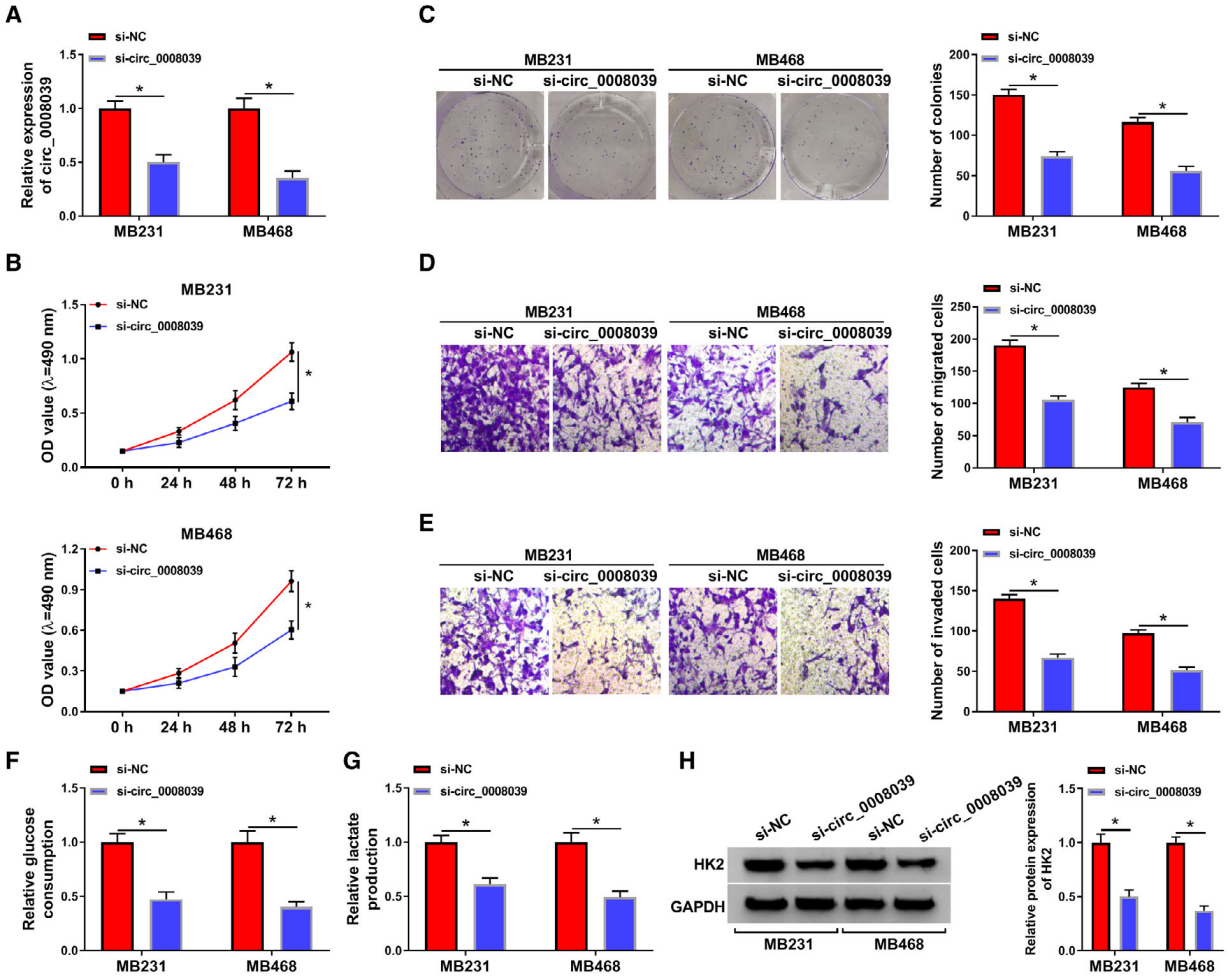
Deficiency of circ_0008039 restrained BC cell proliferation, migration, invasion, and glycolysis. MB231 and MB468 cells were transfected with si‐NC or si‐circ_0008039. (A) The knockdown efficiency of circ_0008039 was examined in MB231 and MB468 cells by qRT‐PCR. (B) Cell viability was evaluated by MTT assay. (C) Colony formation assay was applied to detect colony survival rate. (D and E) Transwell assay was applied for detecting cell migration and invasion. (F and G) Glucose consumption and lactate production were evaluated using commercial kits. (H) HK2 protein abundance was analyzed by western blot assay. The data are shown as the means ± SD of 3 independent experiments. Statistical analysis was conducted using Student’s *t*‐test. *P < 0.05.

To elucidate the role of circ_0008039, MB231 and MB468 cells were transfected with si‐NC (as control) or si‐circ_0008039. The qRT‐PCR analysis was applied for detection of the transfection efficiency of si‐circ_0008039. We noticed that transfection of si‐circ_0008039 evidently reduced the expression of circ_0008039, suggesting the successful introduction of si‐circ_0008039 into MB231 and MB468 cells (Fig. 3A). Next, we measured the effect of circ_0008039 knockdown on cell proliferation. Results revealed that silencing circ_0008039 suppressed cell proliferation by inhibiting cell viability and colony formation in MB231 and MB468 cells (Fig. 3B,C). Transwell assay suggested that silencing circ_0008039 markedly inhibited MB231 and MB468 cell migration and invasion (Fig. 3D,E). Dysregulated cellular metabolism is a typical feature of cancer [19]. Next, we investigated the effect of circ_0008039 downregulation on glycolysis. As illustrated in Fig. 3F,G, knockdown of circ_0008039 inhibited the glucose consumption and lactate production. In addition, silence of circ_0008039 reduced the expression of glucose metabolic enzyme HK2 (Fig. 3H). Thus, we concluded that circ_0008039 silence suppressed BC cell growth, migration, invasion, and glycolysis.

### 3.4 Circ_0008039 was a sponge of miR‐140‐3p in BC cells

**Fig. 4:**
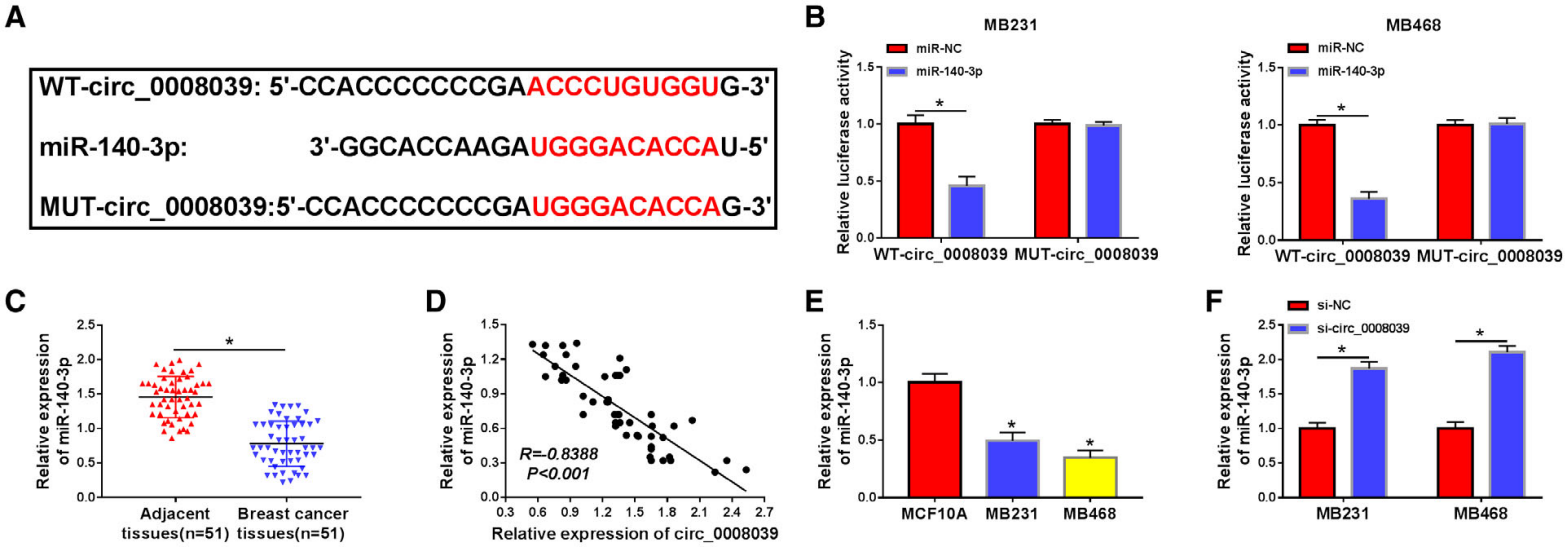
Circ_0008039 acted as a sponge of miR‐140‐3p. (A) The putative binding sites between circ_0008039 and miR‐140‐3p were predicted by circular RNA interactome. (B) The interaction between circ_0008039 and miR‐140‐3p in MB231 and MB468 cells was verified using dual‐luciferase luciferase report assay. (C) MiR‐140‐3p level was measured in BC tissues (*n* = 51) and adjacent normal tissues (*n* = 51). (D) The association between miR‐140‐3p level and circ_0008039 abundance was examined in BC tissues. (E) The expression of miR‐140‐3p was examined in BC cells (MB231 and MB468) and normal human breast epithelial cells (MCF10A). (F) The level of miR‐140‐3p was examined in MB231 and MB468 cells with transfection of si‐NC or si‐circ_0008039. The data are shown as the means ± SD of 3 independent experiments. Statistical analysis was conducted using Student’s *t*‐test. *P < 0.05.

To investigate the underlying mechanism of circ_0008039 function, the potential miRNAs interacting with circ_0008039 were predicted using circular RNA interactome. As presented in Fig. 4A, miR‐140‐3p was predicted as a direct target of circ_0008039. In order to validate this prediction, dual‐luciferase reporter assay was performed in MB231 and MB468 cells. We proved that enforced expression of miR‐140‐3p caused inhibition of luciferase activity in WT‐circ_0008039 group while not in MUT‐circ_0008039 group (Fig. 4B). Moreover, miR‐140‐3p abundance in BC tissues was lower than their normal tissues (Fig. 4C). Furthermore, the relationship between miR‐140‐3p expression and clinical features in BC patients was measured. As shown in Table S2, low level of miR‐140‐3p expression was related to tumor stage, but miR‐140‐3p expression was not related to the age, menopause, tumor size, and lymph node metastasis. Additionally, a distinct inverse correlation between miR‐140‐3p abundance and circ_0008039 level was observed in BC tissues (Fig. 4D). Similarly, we uncovered that miR‐140‐3p was also downregulated in MB231 and MB468 cells relative to MCF10A cells (Fig. 4E). Subsequently, we analyzed the influence of circ_0008039 on miR‐140‐3p expression. The expression of miR‐140‐3p was promoted by interference of circ_0008039 (Fig. 4F). Moreover, we also explored the influence of circ_0008039 on pri‐miR140‐3p and pre‐miR‐140‐3p expression. The data showed that circ_0008039 did not participate in the processing of miR‐140‐3p, and there was no influence between circ_0008039 downregulation and expression of pri‐miR‐140‐3p and pre‐miR‐140‐3p, but only affected the level of mature miR‐140‐3p (Fig. S1A,B). Collectively, miR‐140‐3p could be targeted by circ_0008039 and was negatively regulated by circ_0008039.

### 3.5 Downregulation of miR‐140‐3p reversed the impact of circ_0008039 silence in BC cells

**Fig. 5:**
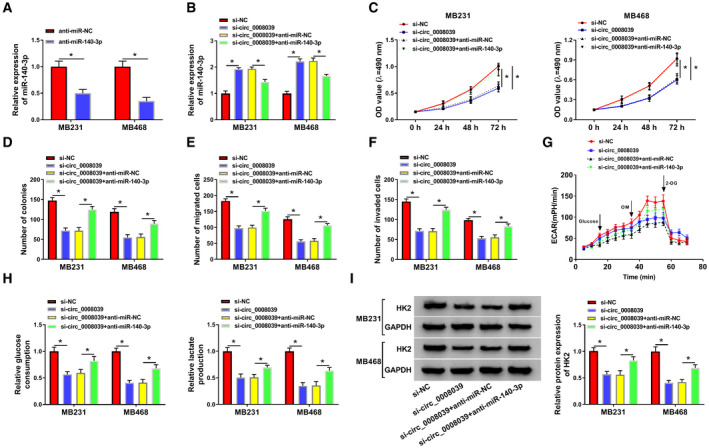
Circ_0008039 exerted its biological functions via sponging miR‐140‐3p in BC cells. (A) Inhibition efficiency of miR‐140‐3p was assessed via qRT‐PCR in MB231 and MB468 cells. (B‐F) MB231 and MB468 cells were transfected with si‐NC, si‐ circ_0008039, si‐circ_0008039 + anti‐miR‐NC, or si‐circ_0008039 + anti‐miR‐140‐3p. (B) The level of miR‐140‐3p was evaluated using qRT‐PCR. (C–F) Cell viability (C), colony formation ability (D), migration ability (E), and invasion ability (F) were determined in MB231 and MB468 cells. (G) ECAR was measured by using Seahorse XF Glycolysis Stress Test kit. OM, oligomycin; 2‐DG, glucose analog 2‐deoxyglucose. (H) Glucose consumption or lactate production was measured using commercial kits. (I) Western blot was conducted to analyze HK2 protein level. The data are shown as the means ± SD of 3 independent experiments. Statistical analysis was conducted using Student’s *t*‐test or ANOVA. *P < 0.05.

Although the relationship between circ_0008039 and miR‐140‐3p was demonstrated, the biological behaviors of BC regulated by circ_0008039 and miR‐140‐3p still needed to be determined. The data displayed that miR‐140‐3p level was reduced by transfection of anti‐miR‐140‐3p in MB231 and MB468 cells (Fig. 5A). Moreover, miR‐140‐3p inhibition counteracted the impact of si‐circ_0008039 on enhancement of miR‐140‐3p expression (Fig. 5B). Meanwhile, silence of miR‐140‐3p partially abated the suppressive influence of si‐circ_0008039 on cell viability and colony formation (Fig. 5C,D). In addition, miR‐140‐3p inhibition attenuated antimigration and anti‐invasion effects caused by interference of circ_0008039 (Fig. 5E,F). Besides, the suppressive effects of circ_0008039 silence on extracellular acidification rate (ECAR) (Fig. 5G), glucose consumption, and lactate production (Fig. 5H) as well as the expression of HK2 (Fig. 5I) were also reversed by downregulating miR‐140‐3p. Hence, we concluded that silencing circ_0008039 suppressed proliferation, mobility, and glycolysis of BC cells via sponging miR‐140‐3p.

### 3.6 SKA2 was a direct target gene of miR‐140‐3p

**Fig. 6:**
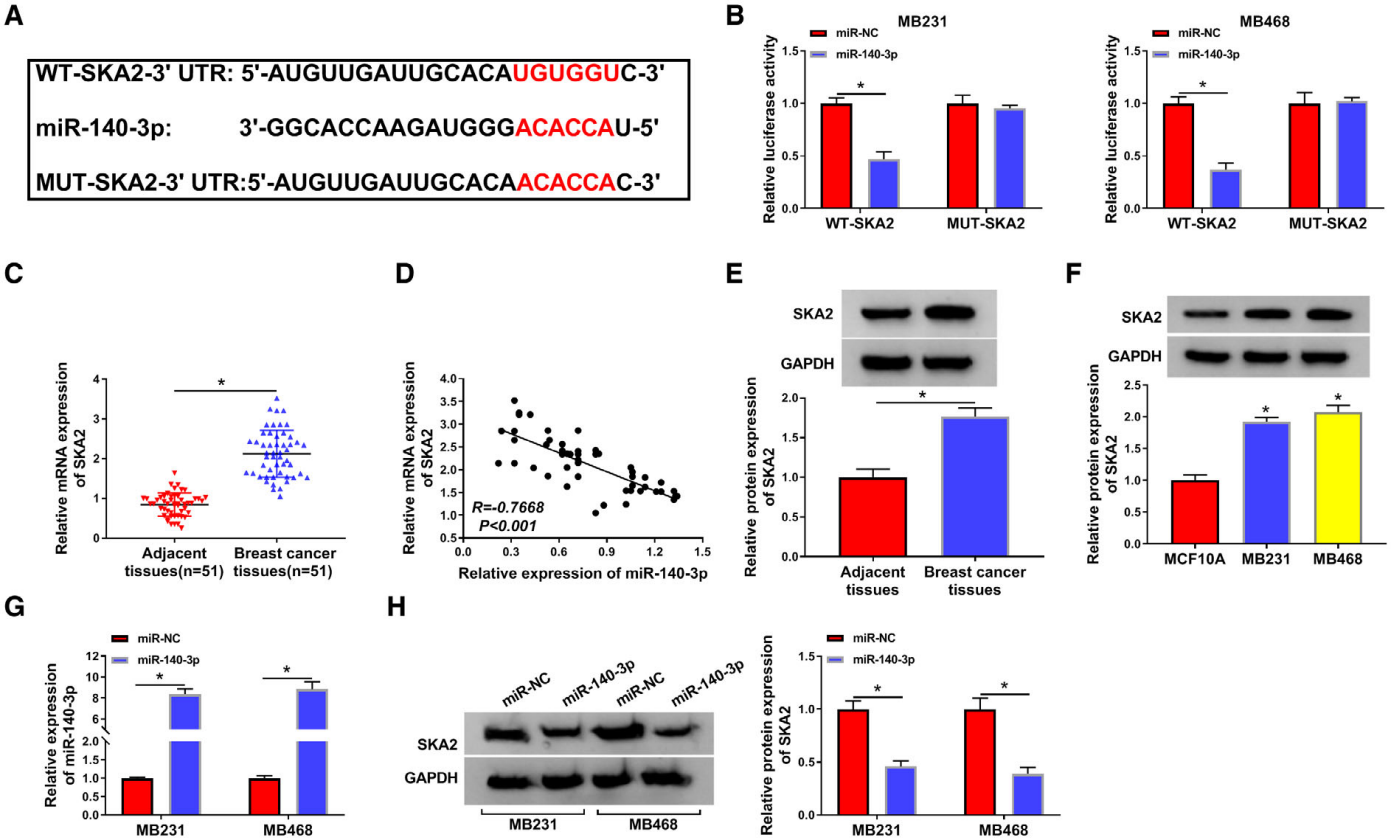
SKA2 directly interacted with miR‐140‐3p in BC cells. (A) The putative targeting sites between miR‐140‐3p and SKA2 were provided by starbase. (B) Relative luciferase activity was detected in MB231 and MB468 cells with cotransfection of WT‐SKA2 or MUT‐SKA2 and miR‐140‐3p or miR‐NC. (C) QRT‐PCR was used for measuring the mRNA expression of SKA2. (D) The correlation between SKA2 and miR‐140‐3p expression was tested in BC tissues. (E and F) The protein level of SKA2 was examined via western blot in BC tissues, BC cells, and their corresponding controls. (G) MiR‐140‐3p expression was examined in MB231 and MB468 cells after transfection with miR‐NC or miR‐140‐3p (H) Western blot was carried out for determination of SKA2 protein level in MB231 and MB468 cells with transfection of miR‐NC or miR‐140‐3p. The data are shown as the means ± SD of 3 independent experiments. Statistical analysis was conducted using Student’s *t*‐test or ANOVA. *P < 0.05.

To clarify the underlying mechanism of miR‐140‐3p, the possible target genes of miR‐140‐3p were predicted by online software starBase. As presented in Fig. 6A, SKA2 3’UTR contained a putative target sequence for miR‐140‐3p, indicating that SKA2 might be a target for miR‐140‐3p. Moreover, enforced expression of miR‐140‐3p apparently suppressed the luciferase activity of WT‐SKA2 in MB231 and MB468 cells, whereas no obvious change was found in MUT‐SKA2 groups (Fig. 6B), confirming that miR‐140‐3p directly targeted SKA2. Furthermore, the mRNA level of SKA2 was distinctly increased in BC tissues relative to adjacent normal tissues (Fig. 6C). In addition, the association between clinical characteristics and SKA2 expression in BC patients was also examined. As illustrated in Table S3, high level of SKA2 was strongly associated with tumor stage, but the level of SKA2 was not associated with age, tumor size, menopause, and lymph node metastasis. And we observed an inverse correlation between the level of miR‐140‐3p and the mRNA expression of SKA2 in BC tissue samples (*R* = −0.7668, *P* < 0.001) (Fig. 6D). Similarly, SKA2 protein level also enhanced in BC tissues and cells (Fig. 6E,F). Moreover, miR‐140‐3p expression was notably increased in MB231 and MB468 cells after miR‐140‐3p transfection (Fig. 6G), implying that miR‐140‐3p was successfully overexpressed. Additionally, the protein expression of SKA2 was inhibited in MB231 and MB468 cells by upregulation of miR‐140‐3p (Fig. 6H). These data proved that SKA2 was a downstream target for miR‐140‐3p and it was inversely correlated to miR‐140‐3p.

### 3.7 SKA2 upregulation weakened anticancer role of miR‐140‐3p in BC cells

**Fig. 7:**
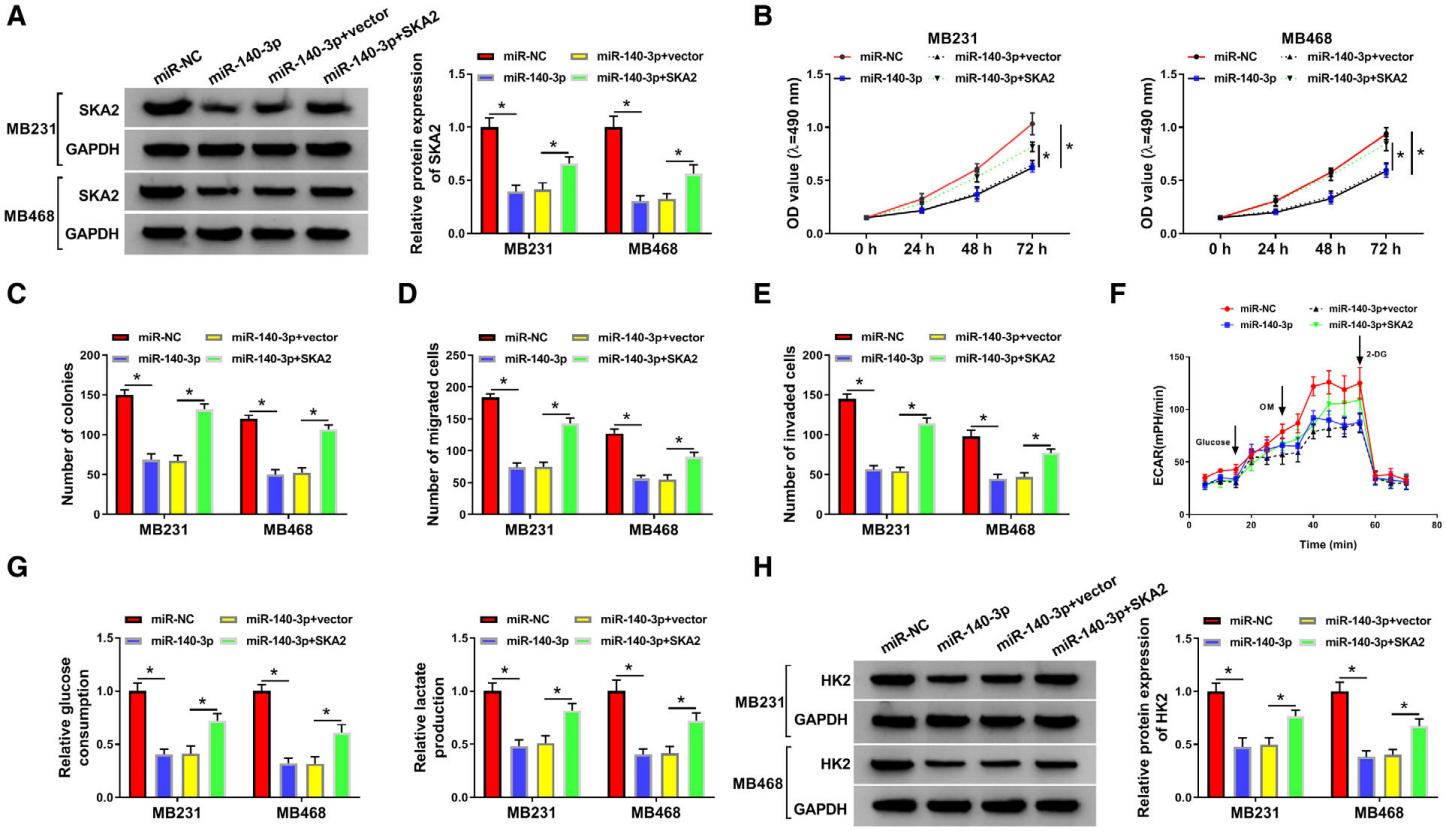
Overexpression of SKA2 weakened the anticancer role of miR‐140‐3p restoration in BC cells. MB231 and MB468 cells were transfected with miR‐NC, miR‐140‐3p, miR‐140‐3p + vector, or miR‐140‐3p + SKA2. (A) Western blot assay determined the protein level of SKA2 in MB231 and MB468 cells. (B–E) Cell viability (B), cell colony‐forming ability (C), migration (D), and invasion (E) were detected in MB231 and MB468 cells. (F) ECAR was measured by using Seahorse XF Glycolysis Stress Test kit. OM, oligomycin; 2‐DG, glucose analog 2‐deoxyglucose. (G) Glucose consumption and lactate production were determined using commercial kits. (H) Western blot analysis measured HK2 protein expression. The data are shown as the means ± SD of 3 independent experiments. Statistical analysis was conducted using Student’s *t*‐test or ANOVA. *P < 0.05.

Next, we explored the function of SKA2 in BC cells. We found that SKA2 was successfully knocked down by transfection of sh‐SKA2 (Fig. S2A). Moreover, downregulation of SKA2 limited cell growth, migration, and invasion of MB231 and MB468 cells (Fig. S2B–E). In addition, SKA2 interference inhibited glycolysis in MB231 and MB468 cells (Fig. S2F–H). To determine whether SKA2 was involved in miR‐140‐3p‐mediated functions, rescue experiments were performed in MB231 and MB468 cells. Results of western blot suggested that overexpression of miR‐140‐3p could inhibit SKA2 expression, which was abated by addition of SKA2 (Fig. 7A). Furthermore, upregulation of SKA2 overturned the repressive influence of miR‐140‐3p restoration on cell viability and colony formation (Fig. 7B,C). At the same time, the repressive effects of miR‐140‐3p on migration and invasion were partially abolished by transfection of SKA2 (Fig. 7D,E). Additionally, accumulation of SKA2 could neutralize the suppressive impact of miR‐140‐3p restoration on ECAR (Fig. 7F), glucose consumption, lactate production (Fig. 7G), and the protein abundance of HK2 (Fig. 7H). Taken together, miR‐140‐3p exerted its anticancer role via downregulating SKA2 expression in BC cells.

### 3.8 SKA2 was modulated by circ_0008039 and miR‐140‐3p in BC cells

**Fig. 8:**
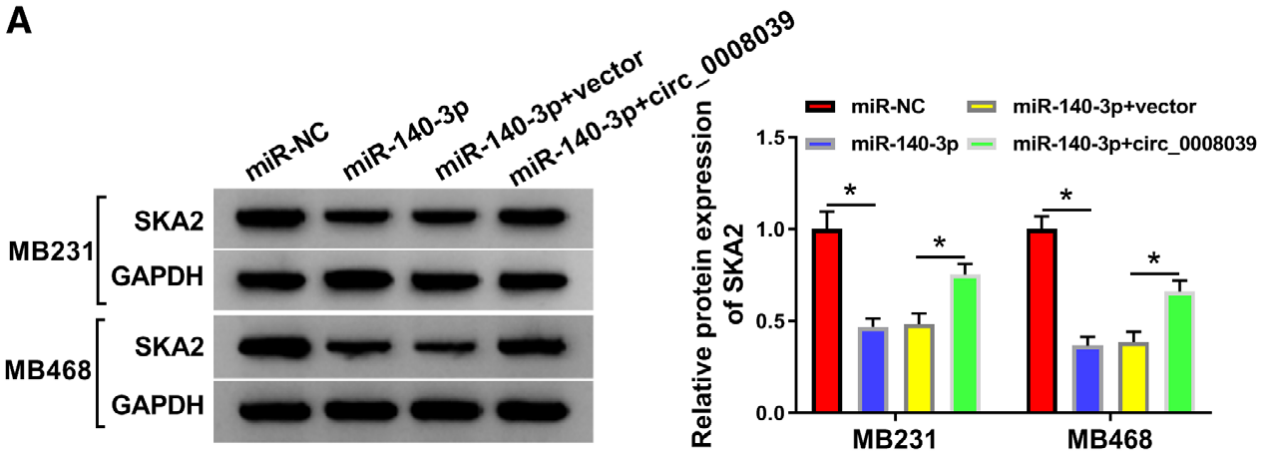
SKA2 expression was modulated by miR‐140‐3p and circ_0008039 in BC cells. (A) SKA2 protein abundance was analyzed by western blot analysis in MB231 and MB468 cells after transfection with miR‐NC, miR‐140‐3p, miR‐140‐3p + vector, or miR‐140‐3p + circ_0008039. The data are shown as the means ± SD of 3 independent experiments. Statistical analysis was conducted using ANOVA. *P < 0.05.

Since miR‐140‐3p was negatively regulated by circ_0008039, we wondered whether circ_0008039 could function as a miR‐140‐3p sponge to affect SKA2 level. The data showed that addition of miR‐140‐3p could suppress SKA2 protein level, whereas the effect was abated by upregulating circ_0008039 (Fig. 8A). Collectively, these data revealed that circ_0008039 could positively regulate SKA2 expression by sponging miR‐140‐3p.

### 3.9 Inhibition of circ_0008039 hampered xenograft tumor growth via modulating miR‐140‐3p and SKA2

**Fig. 9:**
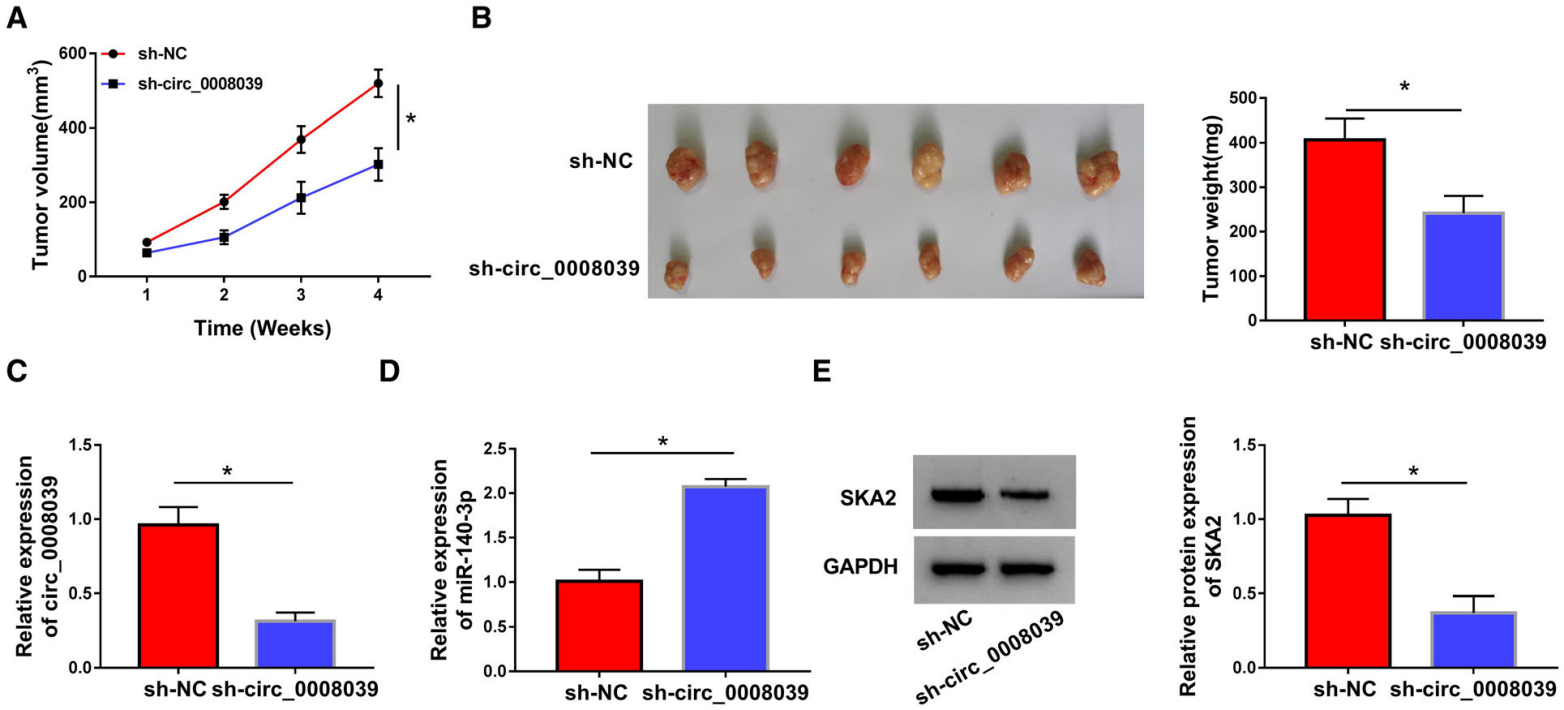
Silence of circ_0008039 suppressed tumor growth via modulating miR‐140‐5p/SKA2 expression. (A and B) The MB468 cells transfected with sh‐circ_0008039 or sh‐NC were inoculated into nude mice, and tumor volume and weight were measured. (C and D) Circ_0008039 and miR‐140‐3p expression levels were examined by qRT‐PCR in resected tumor tissues. (E) SKA2 protein abundance was detected using western blot analysis in resected tumor masses. The data are shown as the means ± SD of 3 independent experiments. Statistical analysis was conducted using Student’s *t*‐test. *P < 0.05.

Finally, we probed the influence of circ_0008039 on tumor growth through an *in vivo* tumorigenesis assay. MB468 cells transfected with sh‐NC or sh‐circ_0008039 were implanted subcutaneously into nude mice. We found that tumor volume and weight were both reduced in sh‐circ_0008039 group with respect to sh‐NC group (Fig. 9A,B). Next, we detected the levels of circ_0008039, miR‐140‐3p, and SKA2 in tumor samples. As presented in Fig. 9C,D, circ_0008039 knockdown resulted in a significant inhibition of circ_0008039 expression and promotion of miR‐140‐3p abundance in collected tumor tissues. Additionally, suppression of circ_0008039 obviously decreased the protein expression of SKA2 in tumor tissues (Fig. 9E). From these data, we demonstrated that interference of circ_0008039 could significantly suppress xenograft tumor growth through increasing miR‐140‐3p and decreasing SKA2.

## 4 Discussion

BC is a common and aggressive malignancy in females. However, the molecular mechanism of BC progression remains largely unexplored. Although miRNAs or lncRNAs have been widely suggested to be involved in the pathogenesis of BC, the involvement of circRNAs in BC development is still to be further investigated. In our research, we found that circ_0008039 downregulation suppressed BC cell proliferation, metastasis, and glycolysis via regulation of miR‐140‐3p and SKA2.

A previous document revealed that circ_0008039 was highly expressed in colon cancer tissues and might be a new prognostic biomarker for the patient with colon cancer [20]. Besides, circ_0008039 abundance was found to be enhanced in both BC tissues and cells, and circ_0008039 promoted BC cell progression through modulating miR‐532‐5p/E2F3 axis [10]. Here, we observed that circ_0008039 level was also elevated in BC tissues and cells, and circ_0008039 expression was positively related to tumor stage and lymph node metastasis, indicating that circ_0008039 might be a critical prognostic biomarker for BC. Moreover, we found that silencing circ_0008039 restrained BC cell proliferation, migration, and invasion. Metabolic alteration was found to be a typical hallmark of tumor cells and most tumor cells primarily rely on aerobic glycolysis to create the energy required for cellular processes [21]. Despite a sufficient supply of oxygen, cancer cells tend to generate energy via cells converting glucose into lactate under aerobic environment, rather than oxidative phosphorylation by mitochondria. This phenomenon, known as the Warburg effect, usually leads to enhanced glucose uptake and lactate production. Next, we investigated the influence of circ_0008039 on glycolysis. Results disclosed that circ_0008039 silence repressed the glycolysis in BC cells. Collectively, these findings implied that circ_0008039 might serve as an oncogene in BC.

It is well known that circRNAs can modulate gene expression via sponging miRNAs in many cancers [22]. Interestingly, we observed that circ_0008039 was mainly localized in the cytoplasm. Given that cytoplasmic cirRNAs mainly serve as miRNA sponges, we then interred that circ_0008039 might function as a sponge of miRNA. By bioinformatics analysis (Circular RNA Interactome) and dual‐luciferase reporter assay, circ_0008039 was found to be a miR‐140‐3p sponge. MiR‐140‐3p was reported to exert the anticancer role in some cancers. For example, miR‐140‐3p could limit lung cancer cell viability and metastasis via decreasing ATP6AP2 expression [23]. Miles *et al*. [24] pointed out that miR‐140‐3p level was reduced in ovarian cancer tumor specimens. Besides, miR‐140‐3p was reported to be downregulated in BC, and miR‐140‐3p restoration could restrain BC cell growth and migration through regulating TRIM28 [15]. Consistent with these results, we demonstrated that the abundance of miR‐140‐3p was decreased in BC cells and tissues, and low level of miR‐140‐3p was closely related to advanced tumor stage. Besides, miR‐140‐3p knockdown could abate the inhibition effect of si‐circ_0008039 in BC process.

It is generally acknowledged that miRNAs commonly exert their roles via directly binding with their target mRNAs [25]. Based on that, starBase was used and presented that there were complementary sites between SKA2 and miR‐140‐3p, and then, the prediction was verified via performing dual‐luciferase reporter assay. It had been reported that dysregulation of SKA2 was strongly related to progression of various cancers [26, 27]. Notably, it has been reported that interference of SKA2 inhibited invasion and metastasis in BC [28]. Also, SKA2 was found to be overexpressed in BC, and downregulation of SKA2 repressed BC cell viability [16]. In this work, SKA2 was highly expressed in both BC tissue samples and BC cells, and high level of SKA2 expression was associated with advanced stage in BC. Moreover, SKA2 knockdown reversed the anticancer role of miR‐140‐3p by increasing BC cell proliferation, mobility, and glycolysis. Furthermore, SKA2 expression could be positively modulated by circ_0008039 and inversely regulated by miR‐140‐3p in BC cells. We further uncovered that silencing circ_0008039 restrained tumor growth *in vivo* through enhancing miR‐140‐3p and decreasing SKA2. Therefore, results showed that circ_0008039 regulated SKA2 by sponging miR‐140‐3p in BC cells.

## 5 Conclusions

In conclusion, we found that circ_0008039 and SKA2 were high expressed and miR‐140‐3p was low expressed in BC. Silencing circ_0008039 inhibited BC cell growth, migration, invasion, and glycolysis partially by upregulating miR‐140‐3p and downregulating SKA2. The circ_0008039/miR‐140‐3p/SKA2 axis is important for the development of molecular targeted therapy for BC.

## Conflict of interest

The authors declare no conflict of interest.

## Author contributions

DD and XW conceived the project. DD and XR designed and performed experiments. SZ, MH, and XX analyzed and interpreted the data. XG and YG wrote the paper. All authors approved final manuscript.

## Supporting information


**Fig S1.** Effect of circ_0008039 on pri‐miR‐140‐3p and pre‐miR‐140‐3p expression.

**Fig S2.** SKA2 knockdown inhibited cell growth, migration, invasion, and glycolysis in breast cancer cells.

**Table S1.** Supplementary Table 1. Association between clinical features and circ_0008039 expression of BC patients (n = 51).
**Table S2.** Association between clinical features and miR‐140‐3p expression of BC patients (n = 51).
**Table S3.** Association between clinical features and SKA2 expression of BC patients (n = 51).
