# Supplementary figures and images for: Circ_0008039 supports breast cancer cell proliferation, migration, invasion, and glycolysis by regulating the miR‐140‐3p/SKA2 axis
Source: Mol Oncol. 2020 Dec 7;15(2):697–709. doi: 10.1002/1878-0261.12862 (PMC7858101; doi:10.1002/1878-0261.12862)

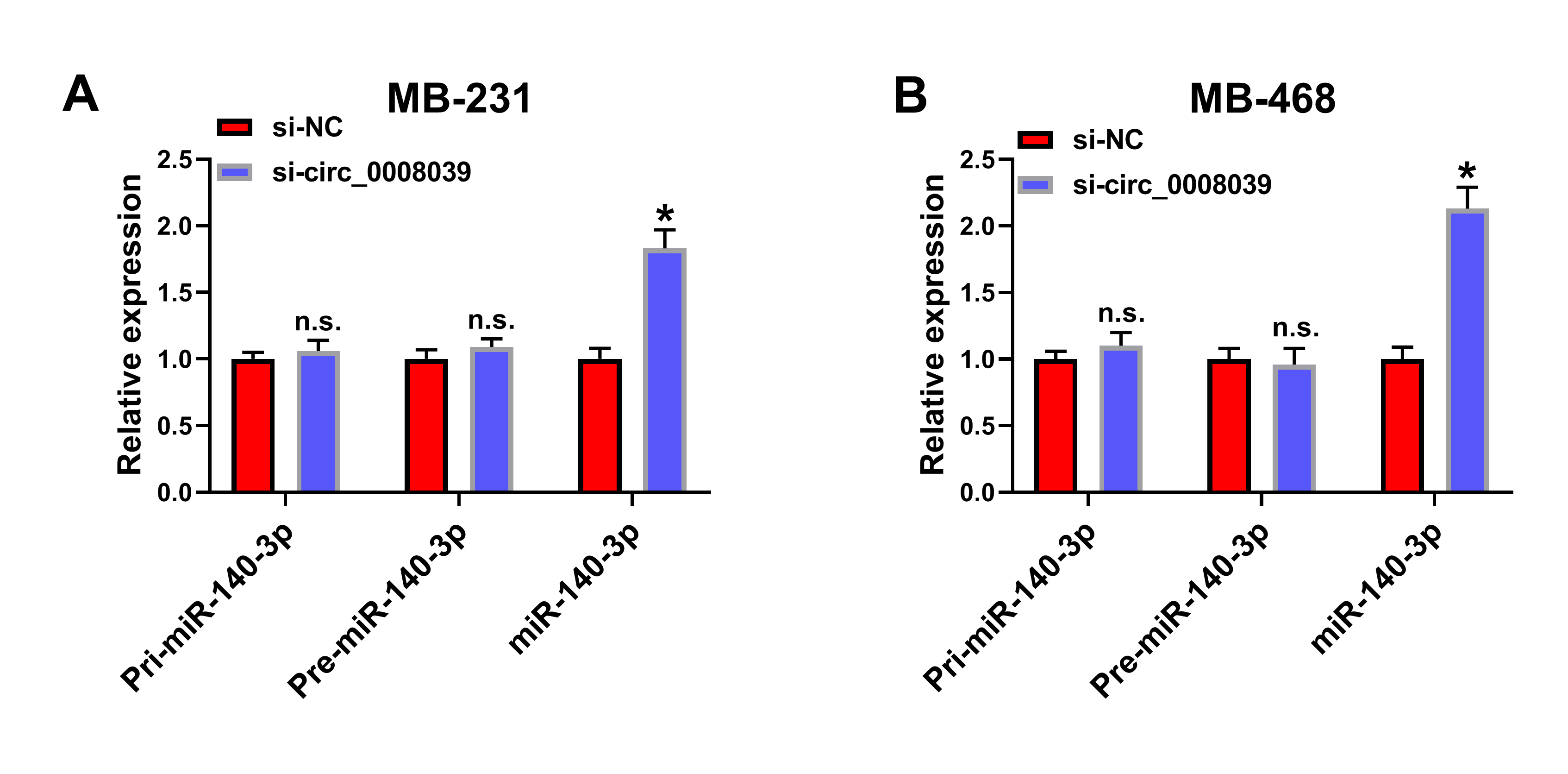

Supplement: Supplementary file 1 — Fig S1. Effect of circ_0008039 on pri‐miR‐140‐3p and pre‐miR‐140‐3p expression. [file MOL2-15-697-s001.tif]

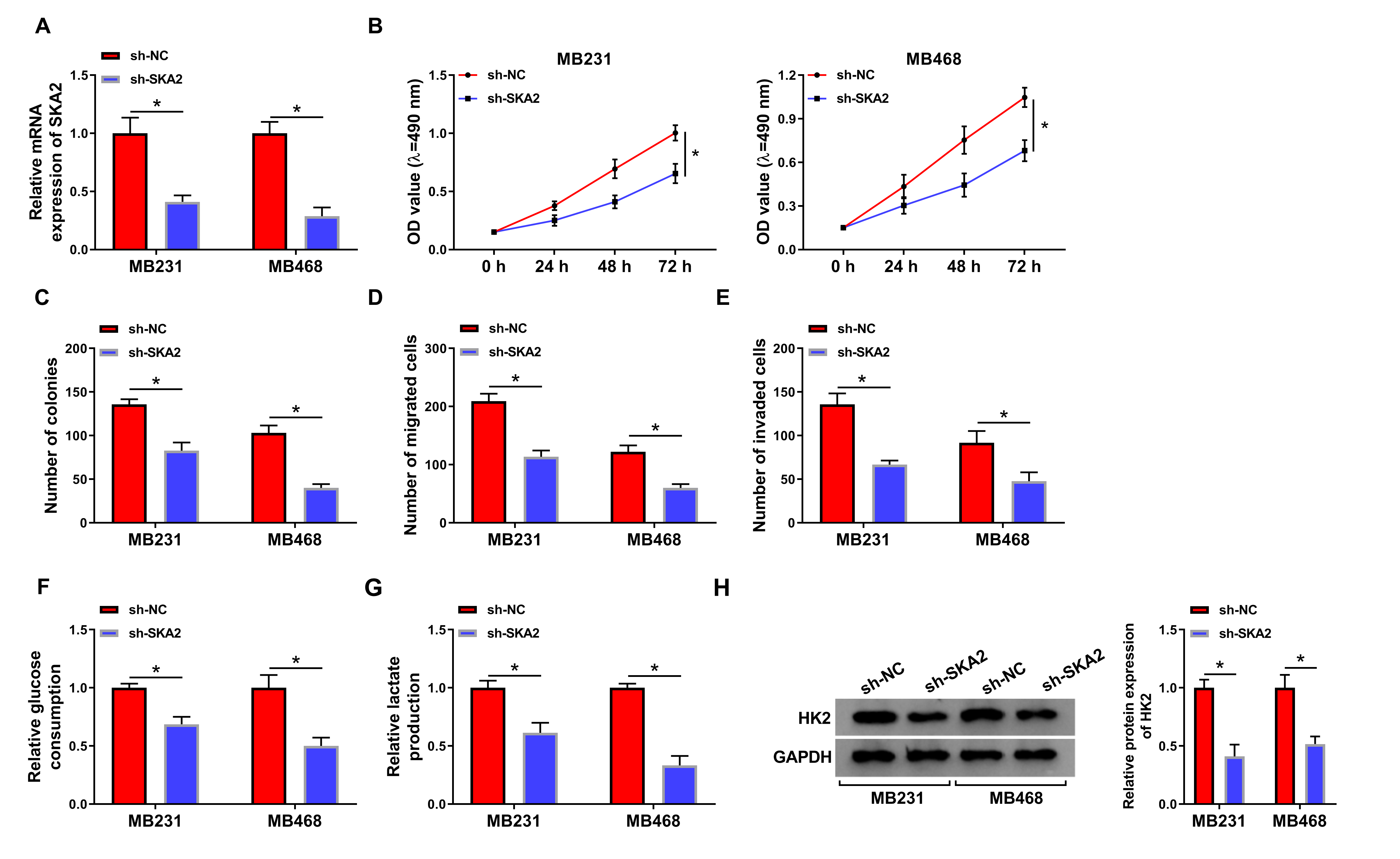

Supplement: Supplementary file 2 — Fig S2. SKA2 knockdown inhibited cell growth, migration, invasion, and glycolysis in breast cancer cells. [file MOL2-15-697-s002.tif]
