# Supplementary material for: Circ_0008039 supports breast cancer cell proliferation, migration, invasion, and glycolysis by regulating the miR‐140‐3p/SKA2 axis
Source: Mol Oncol. 2020 Dec 7;15(2):697–709. doi: 10.1002/1878-0261.12862 (PMC7858101; doi:10.1002/1878-0261.12862)
Supplement: Supplementary file 3 — Table S1. Supplementary Table 1. Association between clinical features and circ_0008039 expression of BC patients (n = 51). Table S2. Association between clinical features and miR‐140‐3p expression of BC patients (n = 51). Table S3. Association between clinical features and SKA2 expression of BC patients (n = 51). [file MOL2-15-697-s003.docx]

Supplementary Table 1. Association between clinical features and circ_0008039 expression of BC patients (n=51)

| Parameter | Case | circ_0008039 expression^a^ | | *P* value |
| --- | --- | --- | --- | --- |
|  |  | High(n=24) | Low(n=27) |  |
| Age (years) |  |  |  |  |
| ≤50 | 25 | 10 | 15 | 0.322 |
| >50 | 26 | 14 | 12 |  |
| Menopause |  |  |  |  |
| No | 23 | 12 | 11 | 0.507 |
| Yes | 28 | 12 | 16 |  |
| Tumor size |  |  |  |  |
| ≤2 cm | 29 | 15 | 14 | 0.444 |
| >2 cm | 22 | 9 | 13 |  |
| Lymph node metastasis |  |  |  |  |
| No | 32 | 11 | 21 | 0.019 * |
| Yes | 19 | 13 | 6 |  |
| Stage |  |  |  |  |
| I+II | 24 | 2 | 22 | 0.0001* |
| III | 27 | 22 | 5 |  |

**P*<0.05,

^a^Using median expression level of circ_0008039 as cutoff

Supplementary Table 2. Association between clinical features and miR-140-3p expression of BC patients (n=51)

| Parameter | Case | miR-140-3p expression^a^ | | *P* value |
| --- | --- | --- | --- | --- |
|  |  | High(n=23) | Low(n=28) |  |
| Age (years) |  |  |  |  |
| ≤50 | 27 | 10 | 17 | 0.220 |
| >50 | 24 | 13 | 11 |  |
| Menopause |  |  |  |  |
| No | 20 | 11 | 9 | 0.254 |
| Yes | 31 | 12 | 19 |  |
| Tumor size |  |  |  |  |
| ≤2 cm | 29 | 14 | 15 | 0.600 |
| >2 cm | 22 | 9 | 13 |  |
| Lymph node metastasis |  |  |  |  |
| No | 32 | 13 | 19 | 0.404 |
| Yes | 19 | 10 | 9 |  |
| Stage |  |  |  |  |
| I+II | 28 | 8 | 20 | 0.009* |
| III | 23 | 15 | 8 |  |

**P*<0.05

^a^Using median expression level of miR-140-3p as cutoff

Supplementary Table 3. Association between clinical features and SKA2 expression of BC patients (n=51)

| Parameter | Case | SKA2 expression^a^ | | *P* value |
| --- | --- | --- | --- | --- |
|  |  | High(n=26) | Low(n=25) |  |
| Age (years) |  |  |  |  |
| ≤50 | 20 | 10 | 10 | 0.910 |
| >50 | 31 | 16 | 15 |  |
| Menopause |  |  |  |  |
| No | 19 | 8 | 11 | 0.329 |
| Yes | 32 | 18 | 14 |  |
| Tumor size |  |  |  |  |
| ≤2 cm | 23 | 12 | 11 | 0.877 |
| >2 cm | 28 | 14 | 14 |  |
| Lymph node metastasis |  |  |  |  |
| No | 24 | 14 | 10 | 0.322 |
| Yes | 27 | 12 | 15 |  |
| Stage |  |  |  |  |
| I+II | 33 | 13 | 20 | 0.025* |
| III | 18 | 13 | 5 |  |

**P*<0.05

^a^Using median expression level of SKA2 as cutoff
